# A Review on Hepatoprotective Effect of Chrysin: Preclinical Implications and Molecular Cascades Came into Focus

**DOI:** 10.2174/0115733998329724240918091335

**Published:** 2024-12-06

**Authors:** Marjan Talebi, Mohsen Talebi, Tahereh Farkhondeh, Jesús Simal-Gándara, Selen İlgün, Ali Mohammad Pourbagher-Shahri, Mohammad Samini, Saeed Samarghandian

**Affiliations:** 1 Department of Pharmacognosy, School of Pharmacy, Shahid Beheshti University of Medical Sciences, Tehran, Iran;; 2 Phytochemistry Research Center, Shahid Beheshti University of Medical Sciences, Tehran, Iran;; 3 Department of Chemistry and Biochemistry, University of Texas at Arlington, Arlington, Texas, 76019, USA;; 4 Department of Toxicology and Pharmacology, School of Pharmacy, Birjand University of Medical Sciences, Birjand, Iran;; 5 Nutrition and Bromatology Group, Department of Analytical and Food Chemistry, Faculty of Science, University of Vigo, Ourense Campus, 32004 Ourense, Spain;; 6 Department of Pharmaceutical Botany, Faculty of Pharmacy, Erciyes University, Kayseri, 38039, Turkey;; 7 Neuroscience Research Center, Mashhad University of Medical Sciences, Mashhad, Iran;; 8 Department of Neurosurgery, School of Medicine, Mashhad University of Medical Sciences, Mashhad, Iran;; 9 Department of Medical Physiology, School of Medicine, Neyshabur University of Medical Sciences, Neyshabur, Iran

**Keywords:** Chrysin, hepatoprotective, flavonoid, cellular and molecular pathways, hepatocellular carcinoma, hepatotoxicity

## Abstract

Chrysin, a flavone nutraceutical, possesses several beneficial pharmacological properties, which has gained much emphasis in recent years. The biological effects of chrysin are exerted due to impeding or activating multifarious cellular and molecular pathways. Our findings indicated that chrysin inhibited tumor progression in various cancer cell lines by repressing the formation of a sphere and upregulated protein expression of Src homology region 2 domain-containing phosphatase-1 (SHP-1), alleviating phosphorylated-signal transducer and activator of transcription 3 (p-STAT3) and transaction workflow innovation standards team1 (Twist1), sustaining phosphorylation of extracellular signal-regulated kinase 1/2 (ERK1/2) and endorsing mitogen-activated protein kinase kinase1 (MEK1) overexpression, increasing the cytochrome c release, mitochondrial reactive oxygen species (ROS) formation, matrix metalloproteinases (MMP) collapse, and caspase-3 activity, modulating p53/ B-cell lymphoma-2 (Bcl-2)/caspase-9 cascade, cyclooxygenase-2 (COX-2), nuclear factor kappa B proposition 65 (NF-κB p65) expression and also decreasing the expression of nuclear factor erythroid 2-related factor 2 (Nrf2). Chrysin prevented cyclophosphamide, doxorubicin, cisplatin, methotrexate, paracetamol, alcohol, carbon tetrachloride, tert-butyl hydroperoxide (tBHP) and thioacetamide. Chrysin has protective properties against oxidative stress, inflammation, hepatotoxicity, liver fibrosis, steatosis, and hepatocellular carcinoma.Chrysin's most common hepatoprotective biochemical and molecular mechanisms involve the ability to control enzyme synthesis, scavenge free radicals, boost the antioxidant response, induce apoptosis, and modify the synthesis of proinflammatory and profibrotic cytokines.Chrysin is a valuable nutraceutical with broad therapeutic feasibility, but to confirm its representative hepatoprotective potential, clinical studies are advised. It would also be interesting to use cutting-edge drug delivery techniques or include bio-enhancers.

## 1 INTRODUCTION

Regardless of the growing advancement in the management of liver disease, these difficulties are still a thought-provoking issue [1]. The liver performs a dominant and integral task in the metabolism and distribution of drugs and nutrients, as well as protection against food xenobiotics [2]. Moreover, the liver participates in maintaining hemostasis, metabolism of glucose and lipids, and formation of hormones [3]. Liver injuries are majorly caused by oxidative stress, which attacks Kupffer cells, endothelial cells, and hepatic stellate cells. Various cytokines such as tumor necrosis factor-alpha (TNF-α) can be generated due to the oxidative stress that affects Kupffer cells, which may cause inflammatory responses and apoptotic fate. Drugs, toxins, alcohol, and environmental pollution are critical inducers of hepatic oxidative damage [4, 5]. Steatosis, steatohepatitis, fibrosis and hepatocellular carcinoma (HCC) most commonly happen attributable to liver damage (Fig. **1**) [6]. Chronic liver disorders, which are accompanied by abnormal regeneration of hepatocytes, unremitting hepatic inflammation, and fibrosis, can result in cirrhosis and genetic/epigenetic changes that cause the formation of dysplastic nodules associated with HCC [7]. About 65% of patients suffering from liver disorders prefer using natural products in the United States and Europe [8].

The discovery of novel drugs is a challenging and multifaceted attempt, with numerous intrinsic complications consisting of the possibility of their synthesis in addition to inherent weaknesses in their pharmacokinetics and toxicity properties. There is a high interest in natural products as drug leads are being revitalized [9, 10]. Besides, nutraceuticals, which are described as animal/plant/microorganism-originated nutritional substances with pharmaceutical applications, are under emphasis at the current time [11]. Chrysin is a flavone secondary metabolite that revealed several expedient biological activities [12].

Regarding preclinical research, chrysin has encouraging protective properties that can be used in the pharmacotherapy of liver disturbances. In the present paper content, we first discussed the general aspects of chrysin. Afterward, we precisely explained its protective effects against various liver disorders by putting our emphasis on molecular mechanisms of action.

## 2 CHEMICAL PROPERTIES, METABOLITES, AND DERIVATIVES OF CHRYSIN

Chrysin is a natural flavonoid compound that could be isolated from various kinds of honey, propolis, plants, and even mushrooms [13, 14] (Table **1**). Chrysin exerts beneficial pharmacological properties including anti-neoplastic, antioxidant, anti-inflammatory, anti-allergic, nephroprotective, hepatoprotective, neuroprotective, effective in reproductive health, anti-diabetic, protective for cardiovascular health, anti-obesity, osteoprotective, anti-dote, and effective against gastrointestinal disorders [15-21] (Fig. **2**). Mechanisms underlying the pharmacological effects of chrysin include modulatory effects on the transcription factors, growth factors, kinases, adhesion molecules, cytokines, autophagy related factors, antioxidants, oxidative stress indices, related enzymes, and apoptosis-related markers [15-21] (Fig. **3**).

The flavone chrysin has three rings; two of them (A and B) are benzene rings, and ring C is a heterocyclic ring that contains oxygen [22]. The presence of a double bond between C2-C3 and the functional group carbonyl on the C4 atom resulted in the antioxidant activities of chrysin [23, 24]. Numerous natural derivatives are established due to the variety of oxygenation in ring-A. Baicalein, oroxylin A, and wogonin are examples of these natural derivatives that originated from chrysin [25, 26]. To ameliorate the pharmacological effects of chrysin, its different derivatives were synthesized, presenting diverse substituents in its molecule [27]. Various chrysin derivatives and analogs can be prepared with optimized biological activities. Alkylation, acetylation, halogenation, methylation, nitration, trifluoromethylation, phosphonation, amine introduction, and organometallic complexion are some of the important chrysin substitutions [28-30]. Gallium-chrysin complexes showed higher anticancer potential associated with more ROS generation [31].

7-aminochrysin derivatives exhibited anti-tumor activities in breast and colon cancer cell lines [32]. Vinylated and allylated chrysin analogs demonstrated prostaglandin E2 (PGE2) inhibitory effects [33]. A hydroxyethyl derivative of chrysin showed anti-inflammatory activities *via* attenuation of nuclear factor kappa B (NF-κB) expression [34]. O-methylation of chrysin led to more reduction in interleukin-6 (IL)-6, monocyte chemoattractant protein-1 (MCP-1), cyclooxygenase-2 (COX-2)-derived PGE2 production, and NF-κB activation [35]. Halogenation of chrysin augmented its affinity to human casein kinase 2 (CK2) [36].

The introduction of fluorine atoms into chrysin is important for enhancing its lipophilicity, solubility, and biological properties, *e.g.*, antibacterial, anticancer, antiviral, and hypoglycemic effects [37].

Fujitaka and coworkers perceived that regioselectively of cultured *Phytolacca americana* cells presented glucosyl and methoxyl residues on chrysin administrated exogenously to provide chrysin 7-β- D- glucoside with more powerful antityrosinase activity and 8- methoxy-chrysin [38]. Another biotransformation of chrysin was performed by using cultured *Eucalyptus perriniana* cells as biocatalysts, which led to the preparation of 7-O-β-D-glucoside and chrysin 7-O-β-gentiobioside [39]. The biotransformation of chrysin by *Cunninghamella elegans* led to the production of chrysin 7-sulfate [40].

About the thermal degradation kinetics and its stability, it was found that chrysin began melting at 558 K, and then evaporation was initiated [41].

Zhou and coworkers found that the solubility of chrysin in ethanol (EtOH) and H_2_O mixtures would rise following an improvement in the concentration of ethanol and an elevation in temperature [42]. Noubigh and colleagues observed that the solubility of chrysin in H_2_O, methanol (MeOH), EtOH, butane-1-ol, butane-2-ol, ethylene glycol, and various mixtures of water+methanol was dependent on an endothermic manner [43].

## 3 PHARMACOKINETICS, BIOAVAILABILITY, AND DRUG DELIVERY SYSTEMS

Chrysin is absorbed inconsiderably, metabolized promptly, and eliminated immediately in the human body. Hence, chrysin has a very low bioavailability [44]. Most commonly, glucuronidation and sulfate conjugation participate in the metabolism of chrysin [45]. Somehow, chrysin is metabolized *via* oxidation in intestinal and hepatic cells [46]. *Nocardia hydrocarbonoxydans* were able to metabolize chrysin by sulfate conjugation, acetylation, and hydroxylation, similar to humans [47].

Dong *et al.* found that sodium oleate-based nanoemulsions of chrysin inhibited the first-pass glucuronidation, which led to great enhancement in the oral absorption of chrysin [48].

Human urine and plasma only contained trace amounts of chrysin conjugated with sulfonate and glucuronide [46], with mice's bile exhibiting the highest concentration of these conjugated metabolites [49]. Consequently, the excretion through feces is the chief recommended gate responsible for the bodily removal of chrysin and its metabolites [25, 46, 49].

Plasma binding >99% was detected following oral intake of a single dose of 400 mg of chrysin in humans [44]. According to evaluations based on Lipinski’s “rule of five,” the oral bioavailability of chrysin was estimated to be about 0.003-0.02% [44, 50], and its maximum plasma concentration was 12-64 nM [51]. Commonly, the expected flavonoid aglycones’ maximum serum concentration is 1 mmol/L [44]. As a result, the chrysin had better be ordered to touch the anticipated micromolar range of serum concentration [27]. Novel drug delivery systems used for the enhancement of bioavailability, solubility, and efficacy of chrysin are stated as liposomes, micelles, and nanoparticles as carriers [52-54]. The best strategy to overwhelm the bioavailability problems of chrysin was employing nanoparticles for the encapsulation of chrysin (Fig. **4**) [55, 56].

Sa *et al.* found that the bioavailability of chrysin in the form of a salt cocrystal of chrysin with berberine is approximately 1.7 times that of pure chrysin in rats [57]. Zhu *et al.* realized that the trapping of chrysin in β-cyclodextrin resulted in an elevation of the solubility of chrysin, its antioxidant potential, anti-tumor effects, and antimicrobial activity [58]. Chrysin-loaded β-cyclodextrin-based nanosponges could improve solubility, drug release, photostability, antioxidant effects, and anti-tumor efficacy of chrysin *in-vitro* [59]. The solid dispersion preparation of chrysin with Brij^®^L4 and amino-clay was apparently encouraging in the enhancement of chrysin dissolution, improving *in vivo* effects as an enhancer of absorption, and promoting the bioavailability of topotecan on human colon cancer cell (HT29) breast cancer cell line [60]. Injectable chrysin-nanoparticles (CH-NPs) exhibited a major delay in non-small cell lung cancer (NSCLC) tumor growth *in vivo*. They offered a lower level of total dosage in comparison to the oral administration of free chrysin [61].

Halevas and coworkers obtained that encapsulating chrysin *via* poly(ε-caprolactone) and poly(3-hydroxybutyrate) microcarriers had valuable hemocompatibility and the potential to combat MDA-MB 231 breast cancer cell line [62]. Fabrication of a delivery platform constructed by the focus on chrysin-polyvinylpyrrolidone sub-micro particles improved its anticancer efficiency [63]. Santos *et al.* found that selenium-chrysin polyurea dendrimer nanoformulation could play a hopeful role in treating ovarian cancer through glutathione depletion and cystathionine β-synthase inhibition in human cell lines [64]. Sassa-deepaeng and colleagues discovered that chrysin-loaded poloxamer micelles improved the water solubility of chrysin, and they were safe regarding the zebrafish embryo growth [65].

Fabrication of metallic silver (Chrysin-AgNPs) and gold (Chrysin-AuNPs) nanoparticles could be a potent approach for breast cancer therapy [66]. L-phenyl alanine-coated iron oxide magnetic NPs could be great carriers for the delivery of chrysin and other hydrophobic agents [67]. Chrysin-anchored Ag and Au NP-reduced graphene oxide composites showed great cytotoxic effects for breast cancer therapy [68]. Gnanasekar and coworkers discovered that the fabrication of reduced graphene oxide nanosheets of chrysin exerted no toxicity in treated animals and also resulted in less lysis of RBCs, which signified their biocompatibility for implication as direct wound dressing [69]. The neuroprotective impact of chrysin NPs against kindling-induced epilepsy might be associated with a reduction of oxidative stress *via* the nuclear factor erythroid 2-related factor/ antioxidant reaction element/transcription factor/Hemoxygenase 1 (Nrf2/ARE/HO-1) signaling pathway [70]. Vedagiri and coworkers obtained that chrysin could be consumed at a lower dose with better oral bioavailability by encapsulating chrysin in solid lipid nanoparticles (SLNs) in Amyloid β25-35 induced Alzheimer’s disease in rats [71]. Chrysin-SLNs molecularly dispersed into the lipids had 5-fold oral bioavailability in comparison to free chrysin and increased permeation into the blood-brain barrier regarding Alzheimer’s disease [72]. Chrysin-bovine serum albumin NPs are a new, compatible, and controlled DDS for cancer therapy [73, 74].

Mohammad *et al.* found that chrysin-curcumin-loaded poly(ε-caprolactone)-poly(ethylene glycol) (PCL-PEG) nano-fibers had great potential for shortening the duration of the wound-healing procedure in rats [75]. Tavakoli *et al.* noticed that nano-encapsulated curcumin-chrysin with a one-step fabricated co-delivery system might increase their efficiency in the treatment of melanoma through augmentation of tissue inhibitor of metalloproteinase (TIMP)-1 and TIMP-2 genes expression and alleviation of metalloproteinases (MMP)-9, MMP-2, and telomerase reverse transcriptase (TERT) genes expression in mouse B16F10 melanoma tumor model [76]. Chrysin-loaded phytosomes, which were prepared by consuming soy phosphatidylcholine or egg phospholipid, could promote glucose uptake [77]. Deldar and coworkers observed that chrysin-loaded PCL/PEG electrospun nanofibrous mats could show feasible enhancements in cell proliferation and adhesion while maintaining the stemness of adipose-derived stem cells [78]. The aforesaid nanofibrous mats alleviated oxidative stress, reduced expression of IL-1β, IL-6, TNF-α, and excessive production of nitric oxide (NO) in J774A1. They maintained the viability of human foreskin fibroblast (HFF)-1 cells subsequent stimulation by lipopolysaccharide (LPS) [79]. Tang *et al.* synthesized novel N-isopropyl acrylamide-based hydrogels with variable content of chrysin multiacrylate, which might have biomedical, environmental, and other applications [80]. Contrary to Staphylococcus aureus, chyrothen-loaded chitosan nanoparticles enhanced antibiofilm activity. This was attributed to the reduction of the hydrophobicity of the cell surface and the synthesis of exopolysaccharide [81].

## 4 TOXICOLOGY PROPERTIES OF CHRYSIN

Chrysin showed an inhibitory effect on human cytochrome (CYP) P450 3A4, which might be accredited to the hydrophobic non-substituted B ring, besides the rigidity of its configuration [82]. Moreover, Chrysin displayed a great inhibitory affinity of 54 nM in the direction of human CYP P450 1A2 [83]. Chrysin and/or its conjugates (chrysin-7-sulfate and chrysin-7-glucuronide) could significantly affect CYP P450 enzymes, Organic anion transporting polypeptides (OATPs), P-glycoprotein (P-gp), breast cancer resistance protein (BCRP), and multidrug resistance-associated protein 2 (MRP2) transporters. Thus, consumption of a high chrysin dose might disturb the drug's transportation and/or biotransformation, which highlights the significance of chrysin dose adjustment in pharmacotherapy regiments [84].

An *in vivo* toxicological study in rats demonstrated that acute oral toxicity (5000 mg/kg) orally exhibited 40% mortality. In the sub-chronic toxicity experiment, treatment with chrysin (1000 mg/kg/day) orally indicated considerable body weight loss, while liver weight was augmented meaningfully in male rats. The lethal dose 50 (LD_50_) value of chrysin was found to be 4350 mg/kg while no-observed-adverse-effect-level (NOAEL) and low-observed-adverse-effect-level (LOAEL) of chrysin were established to be 500 and 1000 mg/kg in that order in male and female rats [85].

Whereas a few quantities of flavonoids are attainable by consuming a regular diet, intake of higher doses of them may induce toxicity commencement [25, 86]. No adverse effects have been reported by intake of 400-500 mg of chrysin per day [87]. The proposed daily dose for consumption of chrysin is well-thought-out to be 0.5-3 g [25, 44]. Chrysin exhibited peroxidase-like properties in hepatocytes, which increased chrysin’s oxidation and the formation of toxins that were related to cytotoxic effects [24]. Topoisomerase II and myeloperoxidase correlated with toxic possessions of chrysin as well [88]. Boosting levels of human testosterone were reported following the consumption of chrysin [89]. Induction of toxicity in trout liver cells and suppression of the formation of *de novo* DNA might be attributed to chrysin intake [24].

## **5.** HEPATOCELLULAR CARCINOMA

Hepatocellular carcinoma (HCC) is one of the most prevailing cancers that can be commenced by heavy alcoholism, obesity/diabetes, nonalcoholic fatty liver disease (NAFLD), NASH, aflatoxin B1, cirrhosis, chronic inflammation in the liver, hepatitis B virus (HBV), and hepatitis C virus (HCV) infections [90-92]. Anciently, the liver has shown great regenerative potential; whether irregular regeneration aggravates or dysplastic nodules form, they are named HCC [93].

According to epidemiological data, only 15% of HCCs have been feasibly treated after invasive surgical operations or organ transplantation. Hence, finding palliative pharmacotherapeutic strategies by synthetic/natural medications to manage this malignancy is very attention-grabbing [94, 95]. FDA-approved systemic therapies for advanced HCC are comprised of multikinase inhibitors (sorafenib, cabozantinib, lenvatinib, and regorafenib), an antagonist of vascular endothelial growth factor receptor (VEFGR2) (ramucirumab), programmed cell death protein 1 (PD-1) pathway targets (nivolumab and pembrolizumab) [91, 96]. Anti-vascular endothelial growth factor A/ immunosuppressive ligand programmed cell death-1 ligand 1 (VEGF/PD-L1) agents may be an emerging immunotherapeutic target in HCC therapy [97].

Abundant mutations have been recognized regarding the occurrence of HCC in humans. The most important mutations entailed in HCC are TERT, tumor protein p53 gene (TP53), Catenin beta-1 (CTNNB1), axis inhibition protein 1 (AXIN1), AT-rich interactive domain-containing protein (ARID) 1A, and ARID2 which have impacts on cell-cycle, telomere maintenance, the Wnt/β-catenin cascade, Janus kinase/signal transducers and activators of transcription (JAK/STAT), reticular activating system /mitogen-activated protein kinase/ Rapidly Accelerated Fibrosarcoma (RAS/RAF/ MAPK), Kelch-like ECH-associated protein 1-nuclear factor (Nrf2/Keap1), and protein kinase B/mammalian target of rapamycin (Akt/mTOR) conduits [93, 98].

Nrf2 knockout is associated with hindering liver regeneration, increasing lesions related to hepatic fibrosis, aggravating hepatic inflammations, and disabling detoxification of hepatic toxins [2]. Nrf2 may play a pivotal part in the angiogenesis process by motivating the expression of hypoxia-inducible factor 1 (HIF-1)α-dependent VEGF in neoplastic cells [99].

Regarding an innovative immunotherapeutic approach in HCC, the transducing growth factor-beta (TGF-β) pathway is feasibly anticipated [91]. P53, which is almost eminent as a tumor suppressor in humans, is the most common gene affected by mutation in tumors and eminently in HCC [100, 101]. HCC therapy, by targeting the gut-microbiota-liver axis, represents feasible opportunities for animals [102].

Iwase *et al.* perceived that after encountering Huh-7 HCC cells with chrysin, the activity of matured structures of sterol-regulating portion-linking proteins has been suppressed, and the generation of *de novo* FAs and cholesterol has been incapacitated [103]. Sherif and colleagues witnessed the glypican 3/ sulfatase 2 (GPC3/SULF2) axis suppressive potential of chrysin in consort with the downregulation of the expression of lncRNA-AF085935 in HepG2 cells [104]. Chrysin expressively repressed the formation of a sphere and upregulated protein expression of SHP-1 in SMMC-7721 and MHCC97H cells. Moreover, chrysin alleviated p-STAT3 and Twist1 expression in SMMC-7721 cells. Together, chrysin is represented as a feasible nomination to combat HCC by regulating the SHP-1/STAT3 pathways [105]. Chrysin inhibited proliferation by increasing the expression of SHP-1, leading to a decrease in STAT3 phosphorylation and, consequently, decreased the expression of cyclin D1, myc1, survivin, and c-myc. The migration and invasion were inhibited by the effect of chrysin on SHP-1/STAT3 pathways *via* a decrease in the MMPs, Rho and Rac expressions. Chrysin also decreased angiogenesis *via* blockage of the SHP-1/STAT3/VEGF pathways (Fig. **5**) [105].

Wei *et al.* discovered that co-treatment of chrysin-sensitized sorafenib *via * ATP-binding cassette superfamily G member 2 (ABCG2) repression. Chrysin provoked sustained phosphorylation of ERK1/2 and endorsed MEK1 overexpression [106]. Chrysin pretreatment was assessed in rats with 2-acetylaminofluorene (2-AAF) and diethylnitrosamine (DEN)-induced HCC. Chrysin administration increased the cytochrome c release, mitochondrial ROS formation, hepatocytes’ mitochondria swelling, MMP collapse, and caspase-3 activity [107]. Chrysin treatment N-nitroso-diethylamine-induced HCC rats alleviated α-fetoprotein levels and the carcino embryogenic antigen [108]. In HCC cells and xenograft animals, chrysin mitigated mitochondrial hexokinase 2 (HK-2) linked with voltage-dependent anion-selective channel 1 (VDAC-1), caused mitochondrial Bax transformation, and induced apoptotic cell death [109]. Chrysin decreased the survival of HCC by increasing the expression of P53, p53 upregulated modulator of apoptosis (PUMA), and phorbol-12-myristate-13-acetate-induced protein 1 (PMAIP1) that stimulated the cytochrome c/caspase 9/caspase 3 cascades leading to apoptosis (Fig. **6**) [110, 111].

Chrysin sensitized the programmed cell fate induced by cisplatin and camptothecin in HepG2. Chrysin also downregulated, B-cell lymphoma-extra large (Bcl-xL), X-linked inhibitor of apoptosis protein (xIAP), functional lumen imaging probe (FLIP), and activated caspase-3 and poly-ADP ribose polymerase (PARP) proteins [112]. Oliveira and coworkers found chrysin stopped the SubG0 phase in the HepG2 cell cycle [113]. Chrysin upgraded TNF-related apoptosis-inducing ligand (TRAIL)-mediated programmed cell death in HepG2 [114]. Gao and coworkers clarified that chrysin impeded the expression of Nrf2 and its downstream genes including MRP5, HO-1, and Aldo-keto reductase family 1 member B10 (AKR1B10) by quenching ERK and phosphatidylinositol-3 kinase (PI3K)-Akt pathway and eventually led to the repeal of doxorubicin-resistant phenotype in BEL-7402/ADM cells [115]. Chrysin increased caspase-3 activity, inhibited VEGF creation, and inhibited angiogenesis in H22 ascitic HCC cells and xenograft mice [116]. Chrysin accelerated the apoptosis induced by TNF-α, the downregulation of apoptosis inhibitory protein cFLIP-L in HepG2 cells. Chrysin elevated the activation of caspase-3, caspase-8, and PARP as well [117].

Sun and coworkers verified overexpression of glucose-regulating protein 78 (GRP78), spliced X-box binding protein-1(XBP-1), and phosphorylation of eukaryotic initiation factor 2 (eIF2-α) by chrysin. Besides, chrysin positively influenced the cleavage of caspase-7 and PARP [118]. Chrysin attenuated COX-2, NF-κB p65 expression, Bcl-xL, and β-arrestin amounts, although it increased mRNA and protein levels of p53, Bax, and caspase-3 in DEN-induced HCC [119]. Smith *et al.* indicated that chrysin’s metabolic constancy would possibly make limitations regarding its capacity to persuade UDP-glucuronosyltransferase 1 (UGT1A1) *in vivo* [120]. Uhl *et al.* found that augmenting the activation of Uridine Diphosphoglucuronosyltransferase (UDGPT) and/or inhibition of sulfotransferase in 2-amino-1-methyl-6-phenylimidazo [4,5-]pyridine (PhIP)-HepG2 might be correlated to the effectiveness of chrysin in hepatoma [121]. Wang and coworkers observed that chrysin nanosuspension could inhibit the proliferation of HepG2 cells and also represented a promising strategy to modify the delivery of chrysin in cancer therapy (Fig. **7**) [122]. Table **2** indicates all studies on the protective effect of chrysin against hepatocarcinoma.

## **6.** DRUGS/TOXINS-INDUCED LIVER DAMAGES

Liver damage caused afterward drug consumption is classified into three groups; (1) direct, (2) idiosyncratic, (3) indirect [123].

The protective effects of chrysin against various toxins and drugs that directly induce liver damage are discussed as the below content (Fig. **8**).

### 6.1 Cyclophosphamide, Doxorubicin, Cisplatin, and Methotrexate

Cyclophosphamide is an alkylating agent from the oxazaphosphorine family which has been useful for the pharmacotherapeutic management of several epithelial tumors, such as ovarian, breast, and small-cell lung carcinomas and hematological carcinomas, such as lymphoma and leukemia. Nevertheless, its usage is restricted because of its adverse effects, mostly hepatoxicity and nephrotoxicity. Cytotoxicity induced hepatorenal toxicity in rats. Treatment with chrysin caused alleviation in hepatic enzymes alanine aminotransferase (ALT), aspartate aminotransferase (AST), and alkaline phosphatase (ALP). Chrysin decreased urea and creatinine as well. Chrysin attenuated MDA and increased superoxide dismutase (SOD), catalase (CAT), glutathione (GSH), and glutathione peroxidase (GPx) in hepatic and renal serum. Inflammatory parameters COX-2, inducible nitric oxide synthase (iNOS), NF-κB, IL-1β, TNF-α, and IL-6 were abridged. Additionally, microtubule-associated protein 1 light chain 3 beta (LC3B) was reduced in chrysin-treated rats. Chrysin decreased degeneration of hepatocytes, hepatocytes’ necrosis, steatohepatitis, hyperemia in sinusoids and vessels, and Bax and increased Bcl-2 expressions [124].

A model of doxorubicin-induced hepatorenal toxicity was studied in rodents by Rashid and coworkers. Chrysin administration diminished the toxicity of biochemical markers of the serum and augmented antioxidant protection enzyme levels, as stated in cyclophosphamide hepatotoxicity [125].

The effectiveness of chrysin in cisplatin-administered rats was investigated. Chrysin alleviated lipid peroxidation (LPO), xanthine oxidase (XO) activity, glutathione depletion, and elevated enzymatic antioxidants CAT, GSH, glucose-6-phosphate dehydrogenase (G6PD), SOD, GPx, and phase-II detoxifying quinone reductase and glutathione S-transferase (GST) activities. Furthermore, chrysin decreased COX-2 and iNOS expression and NF-κB and TNF-α levels [126].

Chrysin protected methotrexate-induced hepatotoxicity in rats through alleviation of ALT, AST, lactate dehydrogenase (LDH) activity, and malondialdehyde (MDA) content in addition to augmentation of GPx, glutathione reductase (GR), SOD, CAT activities, and GST content. Chrysin also reduced Bax, p53, and cleaved caspase-3 [127].

### 6.2 Paracetamol

Paracetamol (acetaminophen) is the most frequently applied antipyretic and analgesic medication universally. Overdose or therapeutic doses of paracetamol cause hepatotoxicity and nephrotoxicity. Glucuronidation and sulfate conjugation are the ways of paracetamol metabolization. It is also metabolized by the cytochrome-P450 system (CYP1A2, CYP 3A4, and CYP2E1), causing N-acetyl-p-benzoquinone imine (NAPQI) formation. Administration of chrysin lessened the NAPQI formation and protected the liver in rats [128, 129]. Chrysin treatment mitigated caspase-3 and LC3B levels, respectively, related to apoptosis and autophagy in the rat model of paracetamol-induced hepatotoxicity [130].

### 6.3 Alcohol

Alcohol is the chief reason for avoidable liver diseases universally. Using alcohol is connected with the incidence and progression of steatosis, alcoholic steatohepatitis, advanced liver fibrosis, cirrhosis, HBV, HCV, and HCC. Alcohol as a liver toxin leads to increased iron deposits, ROS, LPS, cytokines, bile acids, dysbiosis, hepatocyte damage, lipid peroxidation, steatosis, and fibrosis progression [131, 132].

Sathiavelu *et al.* assessed the administration of chrysin to rats with ethanol-induced hepatotoxicity. Chrysin considerably alleviated lipid hydroperoxides, thiobarbituric acid reactive substance (TBARS), and conjugated dienes and suggestively augmented vitamin C and vitamin E, GPx, GR, GST, SOD, and CAT activities, and GSH levels [133]. Tahir and coworkers claimed that the administration of chrysin prevented liver injury induced by chronic ethanol consumption *via* inhibition of antidiuretic hormone (ADH), CYP 2E1, XO, and CAT activities [134].

### 6.4 Carbon Tetrachloride (CCl_4_)

Liver fibrosis represents an overaccumulation of the extracellular matrix (ECM). TGF-β, interleukins, chemokines, NF-κB, PPAR-α and -γ, VEGF, platelet-derived growth factor (PDGF), connective tissue growth factor (CTGF), leptin, tissue inhibitors of metalloproteinases (TIMPs), MMPs, ROS, MCP-1, are believed to form tight junctions between NASH and fibrogenesis [135]. IL-22 has a putative profibrogenic function, which is observed **in vivo* via* the promotion of TGF-β signaling in the condition of a p38/MAPK-reliant mechanism of action [136, 137]. Collagen I is the most plentiful protein in the fibrotic liver. TIMPs are MMP blockers that could lead to cleavage of ECM associated with liver fibrosis [138].

Two studies of CCl_4_-induced liver and kidney injuries in rats were evaluated. Pretreatment with chrysin reduced ALT, AST, creatinine, TNF-α, and MDA levels, and increased SOD and GSH levels. These consequences confirmed that chrysin provided antioxidant activity against CCl_4_-induced hepatic damage [139, 140]. Beyrami and coworkers found that chrysin-loaded nanoliposomes enhanced cadmium-induced hepatotoxicity in mice through the amelioration of iNOS, CAT, SOD, and GPx. Moreover, chrysin-loaded nanoliposomes modulated the liver enzymes and improved the feed intake and body weight gain [141].

CCl_4_-induced acute liver damage in mice was investigated in a recent study. Chrysin administration mitigated TNF-α and alpha-smooth muscle actin (α-SMA) expression [142].

Ciceu *et al.* demonstrated that complexation chrysin with random methyl-β-cyclodextrin and (2-hydroxypropyl)-β-cyclodextrin downregulated NF-κB, TNF-α, and IL-6 gene expression suppressed the activation of hepatic stellate cells, modulated ECM by TIMP-1/MMPs balance, modulated profibrotic and antifibrotic miRNAs expression, downregulated TGF-β1/Smad signaling pathway, mitigated deposition and ultrastructural alterations in CCl_4_-induced liver fibrosis in mice [143]. CCl_4_-induced liver fibrosis in mice [144]. Chrysin exerted suitable efficacy to repress CCl_4_-induced liver fibrosis by inhibition of hepatic stellate cell activation and proliferation *via* the TGF-β1/Smad signaling pathway [145]. Chrysin administration downregulated collagen I and restored TIMP-1/MMP balance [144].

### 6.5 Tert-butyl Hydroperoxide (tBHP)

Huang and coworkers studied tert-butyl hydroperoxide (tBHP)-induced oxidative stress in rat primary hepatocytes. Chrysin upregulated the protein expression of HO-1, ganglion cell layer (GCL), glutamate-cysteine ligase catalytic (GCLC), and glutamate cysteine ligase modifier (GCLM) and increased the intracellular GSH content and GSH/GSSG ratio. Moreover, chrysin attenuated ROS production and activated ERK2/Nrf2/ARE signaling pathways in rat primary hepatocytes [146].

### 6.6 Thioacetamide

Hepatic encephalopathy is a severe neuropsychiatric condition characterized by progressive motor disturbances and declining cognitive function owing to acute or chronic hepatic failure. It is perceived that liver failure has an enduring connection with systemic inflammation. Hence, an increase in brain-measuring levels of TNF-α, IL-1β, and IL-6 following intake of hepato-toxins is the leading cause of hepatic encephalopathy. Targeting this liver-brain axis, which includes systemic pro-inflammatory substances, monocyte recruitment, the gathering of ammonia, manganese, and lactate in the brain, and alteration of blood-brain barrier permeability, probably encourages the process of pharmaceutical care [147]. Moreover, targeting the gut microbiome is another promising approach in hepatic encephalopathy treatment. Fecal microbiota transplantation, glycerol phenylbutyrate, L-ornithine, L-aspartate, polyethylene glycol, and probiotics are novel agents with limited positive evidence for the treatment of hepatic encephalopathy. Probiotics, the same as chrysin, ameliorated neuroinflammatory markers as the aim of therapy [148].

Administration of chrysin in rats toxified with thioacetamide resulted in dampening serum ammonia, AST, and ALT. It also mitigated MDA, elevated GSH, and reduced brain contents of NF-κB, TNF-α, and IL-6. Moreover, chrysin administration reduced the expressions of the TLR-4 gene and caspase-3 protein [149].

## 7 HEPATIC STEATOHEPATITIS AND NONALCOHOLIC FATTY LIVER DISEASE (NAFLD)

Nonalcoholic steatohepatitis is an exceedingly prevalent liver disorder putatively connected with metabolic syndrome and obesity. Recent findings have displayed that NAFLD correlates with the progression of insulin resistance, type II diabetes, hypertriglyceridemia, hypertension, metabolic syndrome, hepatic fibrosis and cirrhosis, and even hepatocellular carcinoma. Hepatocyte injury/fate and numerous genetic/epigenetic/environmental factors are attributed to the pathogenesis of NAFLD.

The presence of type 2 diabetes (T2D) significantly increases the chances of developing NASH and fibrosis compared to NAFLD without T2D. The relationship between NAFLD and T2D is not as straightforward, and these conditions have multiple interactions on different molecular levels, which we tried to summarize in our text. Evidence suggests that NAFLD can precede T2D, so perhaps, by effectively managing NAFLD, we could modify the risk for T2D development in the future.

During T2D, elevation of lipogenesis and reduction of fatty acid oxidation and triglyceride secretion *via* very low-density lipoprotein (VLDL) is found in the liver. In addition, peripheral insulin resistance leads to the release of fatty acid from adipose tissue and an increase in the hepatic uptake of fatty acids (FFAs) [ 150 ]. Therefore, NAFLD and T2D usually coexist. Following metabolic stress, mild liver fat storage initiates a decrease in lipotoxicity. Inflammatory signaling pathways are induced in hepatocytes with high free fatty acid accumulation. In this condition, the NAFLD is stimulated to an advanced stage including cirrhosis and HCC, *via* several molecular mechanisms, including PI3K/ phosphatase and tensin homolog deleted on chromosome 10 (PTEN)/Akt, JAK/STAT, mTOR, 4-hydroxynonenal (4HNE), nuclear respiratory factor-1 (NRF-1) and NF-κB [ 150 ]. The high influx of FFAs liver cells in diabetic conditions causes lipotoxicity and induces oxidative stress and inflammation in the liver, leading to the activation of hepatic stellate cells that promotes fibrosis and the development of HCC [ 150 ]. It seems that changes in gut microbiota (dysbiosis), insulin resistance, lipotoxicity, overnutrition, bile acid metabolism, oxidative stress, apoptosis, autophagy, inflammation, nuclear receptors, innate immunity, and fibrogenesis might be targeted in the treatment of NASH [150-156]. Lifestyle modifications and bariatric surgery are regarded as non-pharmaceutical prudence for the management of NASH. Antioxidant (vitamin E), galectin 3, bovine milk colostrum, fibroblast growth factor (FGF)-19-like substance, FGF-21, peroxisome proliferator-activated receptors (PPAR)-γ agonist (pioglitazone), agonists of PPAR-α and -δ, farnesoid X receptor (FXR) agonist (obeticholic acid), antagonists of chemokine receptor-2 and 5, the inhibitor of lysyl oxidase-like 2, and the inhibitor of stress-activated kinase-1 are pharmacotherapies in phase 2/3 of clinical trials for NAFLD [157-159]. Jointly, the best therapeutic attitude for NAFLD should be multitargeted and holistic [160].

Methionine and choline deficiency (MCD) caused NAFLD in mice, and H_2_O_2_-induced damage in HepG2 cells was assessed. Treatment with chrysin caused a reduction in liver triacylglycerol. Furthermore, attenuation in dihydroethidium (DHE) fluorescence, MDA, and an increase in SOD, CAT mRNA, and protein expressions were observed. Chrysin augmented hepatic VLDL secretion by apoprotein (Apo)B upregulation and attenuated hepatocyte Nuclear Factor 4 (HNF4)-α at ser78 in NAFLD mice. Besides, chrysin inverted the consequence of oxidative damage, which was correlated to a decrease in expression of ApoB and secretion of VLDL **in vitro*.* Additionally, chrysin lessened protein kinase C (PKC) performance in MCD mice liver [161]. Chrysin administration attenuated serum fasting glucose and improved insulin resistance, as well as decreased liver enzymes and improved dyslipidemia in rats fed by HFD. Chrysin demonstrated antioxidant properties, abridged hepatic concentrations of carbonyl, advanced glycation end products (AGEs), IL-6, TNF-α, and collagen. Chrysin considerably alleviated the hepatic sterol regulatory element-binding protein (SREBP)-1c gene expression and augmented its attributable PPAR-α [162].

## CONCLUSION

Chrysin, a promising bioflavonoid, is consumed for various therapeutic purposes. Regarding data provided in this review study, the bioavailability of chrysin can be enhanced by nanocarriers and its activity can be promoted by the synthesis of more efficient derivatives. Chrysin showed emerging hepatoprotective properties in preclinical studies through scavenging free radicals, anti-inflammatory responses, inducing apoptotic cell death, regulation of enzymes and antioxidants, numerous transcriptional signaling pathways, and modulation of biochemical changes. Findings and discussion of our study demonstrated that chrysin possesses great hepatoprotective properties to combat hepatotoxicity, liver fibrosis, NAFLD, hepatic encephalopathy, and hepatocellular carcinoma by evidence of preclinical studies. The protective effects of chrysin against liver cancer are mostly related to its effects on the SHP-1/Stat3, Sulf2/GPC3/ wingless and Int-1(Wnt)/B-Catenin, and P53/PUMA or phorbol-12-myristate-13-acetate-induced protein 1 (PMAIP1) /Bcl2/Bax/cytochrome c/caspase9/caspase3. In addition, its inhibitory effects on Nrf2, Erk1/2 and PI3K lead to an increase in oxidative stress in tumor cells, which leads to a decrease in tumor cell survival and proliferation. Chrysin can mostly ameliorate the hepatotoxicity induced by drugs and chemicals *via* inhibition of NF-κB, ROS, p53, and LC3B, as well as its stimulatory effects on the Nrfs signaling pathways. In addition, chrysin is able to modulate fibrogenesis induced by drugs and chemicals *via* a decrease in the α-SMA, ECM, collagen 1, and TGF-B1/Smad. Despite all the worthwhile characteristics of chrysin for liver protection, evaluation of its promising effects in clinical trials is highly recommended for prospects.

## AUTHOR'S INSIGHT ON THE TOPIC

To the best of our knowledge, phytochemicals, nutraceuticals, and herbal medicines have feasible therapeutic effects in prevailing liver disorders.

With regard to the promising hepatoprotective impact of chysin in hepatocellular carcinoma, fatty liver diseases, and hepatotoxicity in preclinical studies, this grateful nutraceutical can be evaluated *via* efficient clinical trials.

It’s possibly evident that the application of nanotechnology-based drug delivery systems can assist in the enhancement of the bioavailability of chrysin. Somehow, phytosomal formulations are of high interest due to the synergistic efficacy observed by the presence of phosphatidylcholine in the preparation.

Besides the previous recommendations in the targeted issue, pharmacovigilance and post-marketing adverse drug reactions should be assessed in future investigations.

## MAIN POINTS

• Chrysin has protective properties against hepatotoxicity, liver fibrosis, steatosis, and hepatocellular carcinoma.

• The most hepatoprotective mechanisms of chrysin include the potential for regulating enzymes, scavenging free radicals, antioxidant response, modulating the synthesis of proinflammatory and profibrotic cytokines, and inducing apoptosis.

• Chrysin may be a valuable nutraceutical agent that can be used in novel drug delivery systems.

## Figures and Tables

**Fig. (1):**
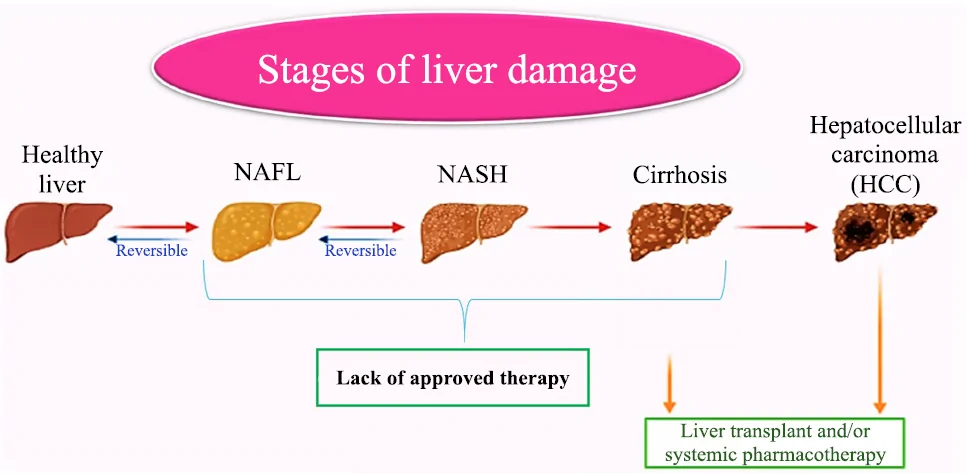
Alterations in the liver and the therapeutic strategies in each stage.

**Fig. (2):**
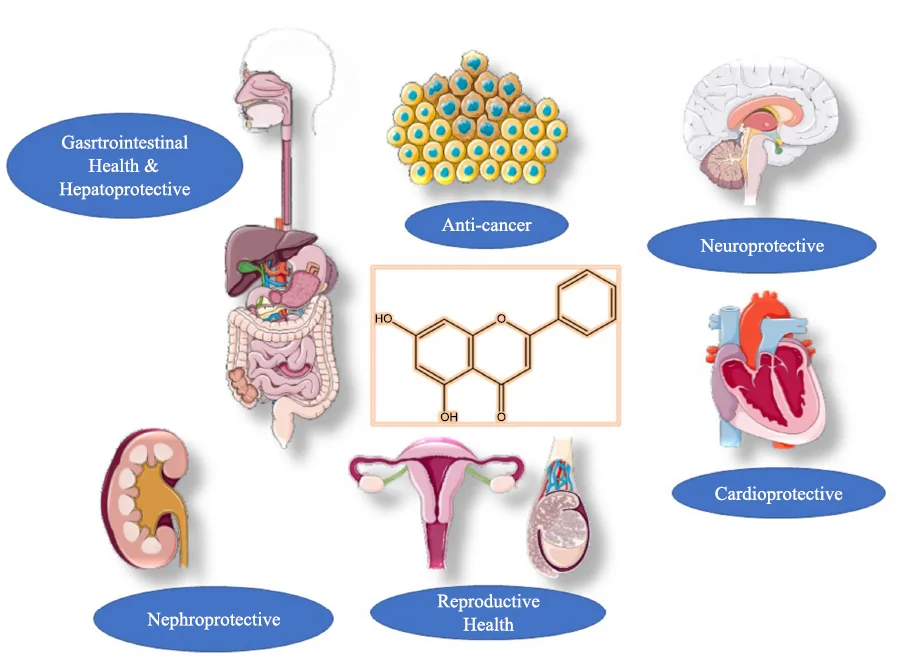
Protective and therapeutic indications of chrysin in numerous diseases.

**Fig. (3):**
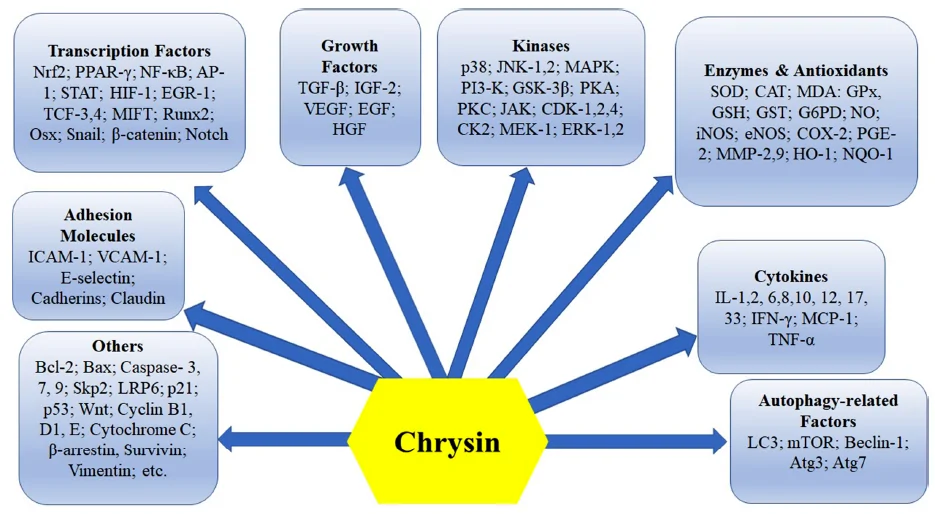
Pharmacological effects of chrysin concerning mechanisms of action. **Abbreviations: **Nrf2: Nuclear erythroid 2-related factor 2; PPAR-γ: peroxisome proliferator-activated receptor gamma; NF-κB: nuclear factor kappa-light-chain-enhancer of activated B cells; AP-1: activator protein 1; STAT: signal transducer and activator of transcription; HIF-1: hypoxia-inducible factor 1 alpha; EGR-1: Early growth response protein 1; TCF-3, 4: Transcription factor 3,4; MIFT: Microphthalmia-associated transcription factor; RUNX2: Runt-related transcription factor 2; Osx: Osteoblast-specific transcription factor Osterix; SOD: superoxide dismutase; CAT: catalase; GPx: glutathione peroxidase; GSH: glutathione; GST: glutathione-S-transferase; HO-1: heme oxygenase-1; NQO1: NAD(P)H:quinone oxidoreductase 1; COX-2: cyclooxigenase 2; iNOS: inducible nitric oxide synthase; eNOS: endothelial nitric oxide synthase; MMP: matrix metalloproteinase; PGE-2: prostaglandin E2; NO: nitric oxide; G6PD: glucose-6-phosphate dehydrogenase; IL-10: interleukin-10; IFN-γ: interferon gamma; MCP-1: monocyte chemoattractant protein 1; TNF-α: tumor necrosis factor alpha; LH-3:Microtubule-associated protein 1A/1B-light chain 3; mTOR: mechanistic target of rapamycin; Beclin-1: mammalian orthologue of yeast Atg6; Atg: autophagy-related; TGF-β: transforming growth factor beta; HGF: hepatocyte growth factor; IGF-2: insulin-like growth factor 2; VEGF: vascular endothelial growth factor; EGF: endothelial growth factor; ICAM-1: intercellular adhesion molecule-1; VCAM-1: vascular cell adhesion molecule-1; JNK: c-Jun NH(2)-terminal kinase; MAPK: mitogen-activated protein kinase; PI3-K: phosphoinositide 3-kinase; GSK-3β: glycogen synthase kinase 3 beta; PKA: protein kinase A; PKC protein kinase C; JAK: Janus kinase; CDK: cyclin-dependent kinase; CK2: casein kinase 2; MEK-1: Mitogen-activated protein kinase 1; ERK-1, 2: extracellular signal-regulated protein kinases 1,2.

**Fig. (4):**
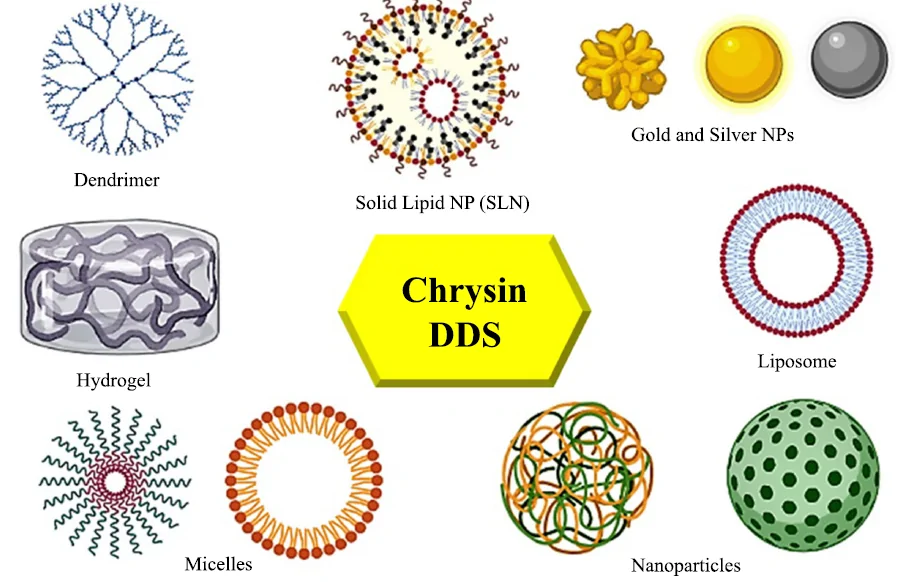
Novel drug delivery systems of chrysin. **Abbreviations: **DDS: drug delivery system; NP: nanoparticle; SLN: solid lipid nanoparticle.

**Fig. (5):**
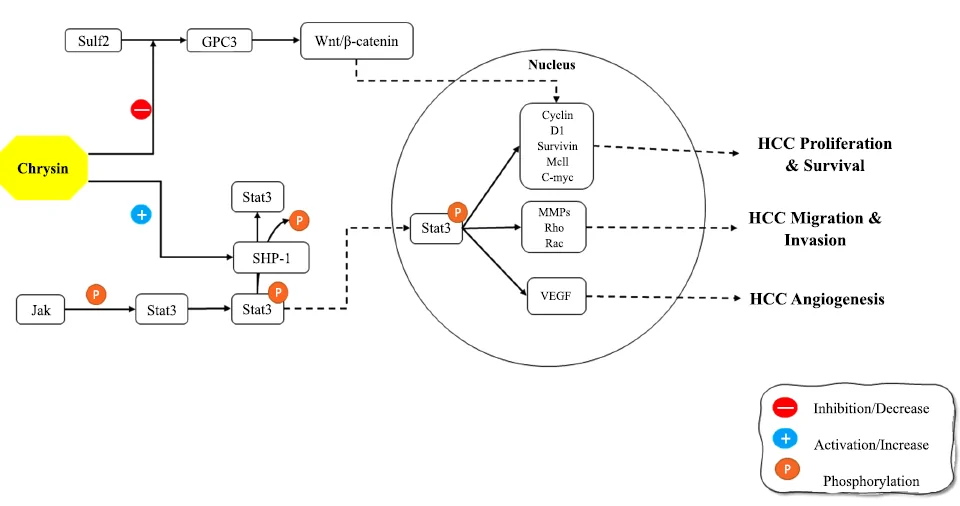
Effects of chrysin on cellular pathways and targets related to development and invasion of HCC. **Abbreviations: **HCC: Hepatocellular Carcinoma; JAK: Janus kinase; STAT: JAK-signal transducer and activator of transcription; SULF2: Sulfatase 2; GPC3: Glypican 3.

**Fig. (6):**
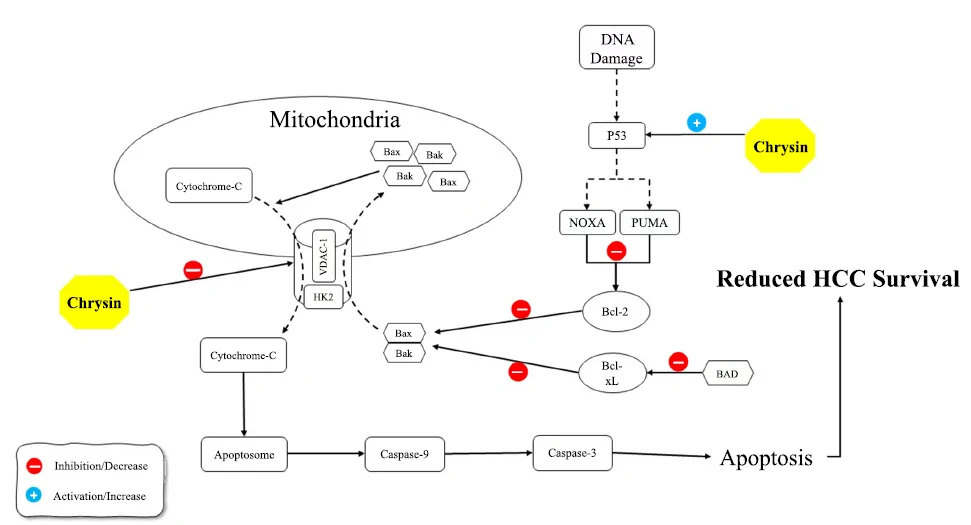
Effects of chrysin on cellular pathways and targets related to development and invasion of HCC. **Abbreviations: ** HCC: Hepatocellular Carcinoma; Bcl-2: B-cell lymphoma 2; VDAC-1: Voltage-dependent anion-selective channel 1; HK2: Hexokinase 2.

**Fig. (7):**
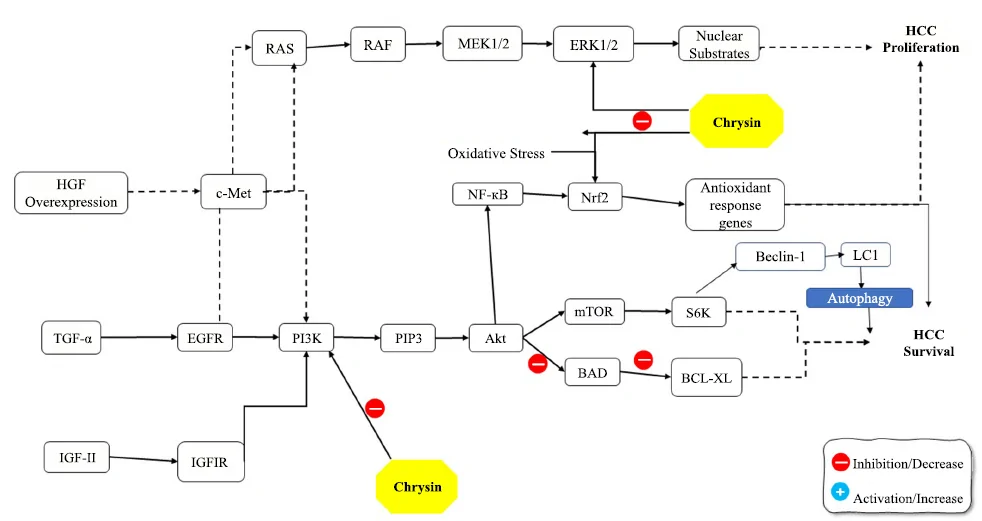
Effects of chrysin on cellular pathways and targets related to development and invasion of HCC. Chryisn can decrease HCC survival through induction apoptosis, autophagy andinhibition oxidative stress. Chrysin can induce autophagy by inhibiting PI3K/Akt/mTOR/SK6/Beclin-1/LC1 signaling pathway. The apoptosis can be activaited by chrysin treatment through inhibiting PI3K/AKt/BAD/BCL-XL signaling pathway. Chrysin can suppress cellprolifration through inhibiting PI3K/ RAS/RAF/MEK1/2/ERK1/2 and PI3K/ Akt/ NF-κB/Nrf2 signaling pathways. **Abbreviations: ** HCC: Hepatocellular Carcinoma; HGF: hepatocyte growth factor; c-Met: tyrosine-protein kinase Met or hepatocyte growth factor receptor (HGFR); EGFR: epidermal growth factor receptor; TGF-α: Transforming growth factor-alpha; IGF-II: insulin-like growth factor; IGFR: insulin-like growth factor receptor; PI3K: phosphatidylinositol-3 kinase; PIP3: phosphatidylinositol-3,4,5-triphosphate; NF-κB: Nuclear factor-kappa (B). mTOR: mechanistic target of rapamycin; Nrf2: nuclear factor erythroid 2-related factor 2; ERK: extracellular regulated MAP kinase.

**Fig. (8):**
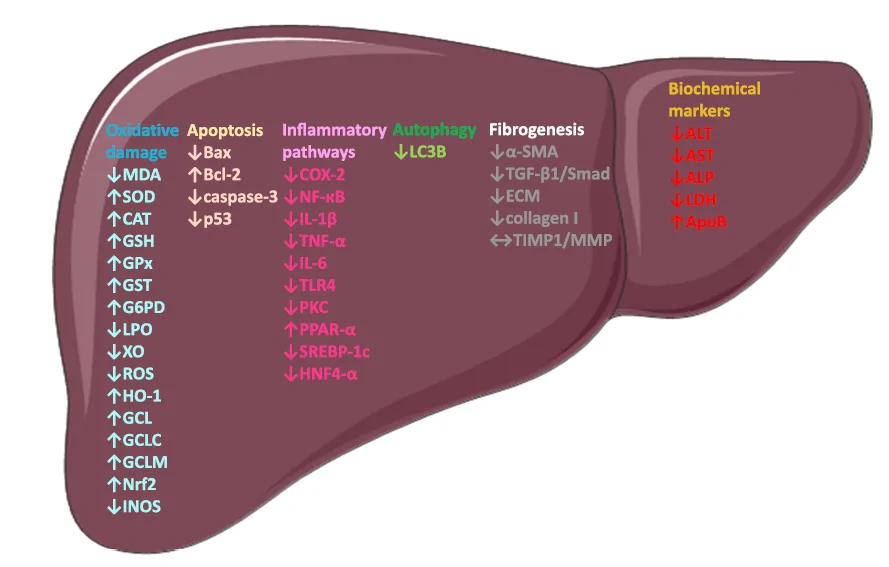
Molecular pathways involved in protective effects of chrysin against drugs/toxins-induced hepatotoxicity.

**Table 1:** Sources of chrysin.

**Botanical Name**	**Family**	**Type**	**Used Part**	**Extract**	**Isolation Technique**	**Yield**	**References**
Croatian Propolis	-	-	-	-	HPLC/UV-Vis	2478.5 µg chrysin /g propolis	[163]
Iraqi Propolis	-	-	Collected by honeybees (*Apis mellifera)*	-	HPLC-ESI/MS	10-1505 µg chrysin/mL propolis	[164]
Buckwheat kinds of honey	-	-	Nectar of little pink flowers collected by honeybees	-	HPLC-DAD/ESI-MS	19.2-128.2 µg chrysin/100 g honey	[165]
Acacia honey	-	-	-	-	HPLC	-	[166]
*Pelargonium crispum*	Geraniaceae	Plant	Leaves	Me_2_CO	HPLC/MS	-	[167]
*Desmos cochinchinensis*	Annonaceae	Plant	Leaves	CH_2_Cl_2_: MeOH (9.5: 0.5)	Preparative TLC/UV-Vis (FTIR)	10.5 mg chrysin/774 g dried leaves	[168]
*Scutellaria araxensis*	Lamiaceae	Plant	Roots, shoots	MeOH	HPLC/UV-Vis	Root: 38.14 µg chrysin/ 1.25 g dried roots Shoot: 235.5 µg chrysin/ 1.25 g dried shoots	[169]
*S. bornmuelleri*	Lamiaceae	Plant	Roots, shoots	MeOH	HPLC/UV-Vis	Root: 100.56 µg chrysin/0.4 g dried roots Shoot: 144.94 µg chrysin/0.26 dried shoots	[169]
*S. immaculata*	Lamiaceae	Plant	Aerial parts, roots	MeOH, CHCl_3_, Water	HPLC/ESI-MS	-	[170]
*S. lateriflora*	Lamiaceae	Plant	Aerial parts	MeOH	HPLC-DAD/ESI-MS	-	[171]
*S. multicaulis*	Lamiaceae	Plant	Roots, shoots	MeOH	HPLC/UV-Vis	Root: 26.63 µg chrysin/ 1.25 g dried roots Shoot: 13.48 µg chrysin/ 2.5 g dried shooots	[169]
*S. ramosissima*	Lamiaceae	Plant	Aerial parts, roots	MeOH, CHCl_3_, Water	HPLC/ESI-MS	-	[170]
*S. virens*	Lamiaceae	Plant	Roots, shoots	MeOH	HPLC/UV-Vis	Root: 144.96 µg chrysin/0.135 g dried roots Shoot: 173.98 µg chrysin/0.2 g dried shoots	[169]
*Oroxylum indicum*	Bignoniaceae	Plant	Roots	MeOH (70%)	HPTLC/UV-Vis	-	[172, 173]
*Cytisus villosus*	Fabaceae	Plant	Aerial parts	EtOH (80%) (EtOAc fraction)	Preparative TLC/UV-Vis (NMR)	4 mg chrysin/1000 g dried aerial parts	[174]
*Cytisus multiflorus*	Fabaceae	Plant	Flowers	EtOH (80%)	HPLC-DAD/ESI-MS (NMR)	0.5 mg/g dried plant	[175]
*Eriodictyon californicum*	Hydrophylloideae	Plant	Twigs and leaves	EtOH (95%)	Column and plate chromatography/UV (NMR)	2 mg chrysin/800 g plant	[176]
*Passiflora coerulea*, *P. incarnata*	Passifloraceae	Plant	-	-	-	-	[177, 178]
*Morinda citrifolia*	Rubiaceae	Plant	Fruits	Hexan, CHCl_3_, EtOAc, EtOH, MeOH	HPLC	-	[179, 180]
*Docynia delavayi*	Rosaceae	Plant	Rhizomes	MeOH: Water (9:1)	HPLC/ UV, IR, MS, NMR	-	[116, 181]
*Pyrus pashia*	Rosaceae	Plant	Fruits	EtOH (95%) (EtOAc fraction)	Column chromatography/NMR, LC-MS	-	[182]
*Crataegus oxyacantha*	Rosaceae	Plant	Twigs	MeOH	Column chromatography/UV (NMR)	-	[183]
*Mitrella kentii*	Annonaceae	Plant	Leaves	Hexan, EtOAc, MeOH	Column chromatography/ NMR, ESI-MS	-	[184]
*Mangifera indica*	Anacardiaceae	Plant	Leaves	MeOH	GC-MS, HPTLC	-	[185]
*Alpinia oxyphylla*	Zingiberaceae	Plant	Fruits	EtOH (80%)	HPLC- QTRAP	-	[186, 187]
*Chaetomium globosum* PG 1.6	Chaetomiaceae	Fungus	Isolated from a marine green alga *C. media*	Fermentation, extraction by EtOAc	GC-MS, TLC-UV-Vis (FTIR, NMR), LC-MS	-	[188]

**Table 2:** The protective effect of chrysin in hepatocellular carcinoma.

**Experimental Model**	**Dose and Duration of Treatment**	**Molecular Mechanism**	**References**
Huh-7 cells	100 µM24 h	Decreased the expression of SREBP target genes *via* the degradation of SREBPs mature forms	[103]
HepG2	15, 30, and 60 μg/mL 24/48 h	Suppressed the GPC3/SULF2 axis along with the downregulation of lncRNA-AF085935 expression	[104]
SMMC-7721 and MHCC97H cells	10 Mm/L	Suppressed SHP-1/STAT3 signaling axis	[105]
Hep3B and HepG2	25 μM 48 h	Sustained phosphorylation of ERK1/2	[106]
Hepatocellular carcinoma (HCC) rat model	10, 20, and 40 µM	Increased in mitochondrial reactive oxygen species (ROS) generation, Collapsed the mitochondrial membrane potential (MMP), Swelled mitochondria, and cytochrome c release	[107]
N-nitrosodiethylamine-induced hepatocellular carcinoma in rats	50 mg/kg 16 weeks	Blocked oxidative stress indices, Decreased the expression of PCNA protein	[108]
HCC cell line HepG2, Hep3B, Huh-7, HCC-LM3, Bel-7402 and SMMC-7721	30 and 60 µM 24, 48 and 72 h.	Declined HK-2 combined with VDAC-1 on mitochondria resulted in the transfer of Bax from cytoplasm to mitochondria and induction of cell apoptosis	[109]
HCC cell xenograft model	30 mg/kg	Decreased HK-2 expression	[109]
HepG2 and QGY7701 cells	0, 10, 15, 20, 25, 30, 40 and 50 µg/ml 24 h	Regulated the p53/Bcl-2/caspase-9 signaling pathway	[110]
HepG2 cells.	pretreated with chrysin (0, 10,20 and 40 µM) for 2 h	Increased the phosphorylation and accumulation of p53 through activating ERK1/2 overexpression of the pro-apoptotic proteins Bax and DR5 and the inhibition of the anti-apoptotic protein Bcl-2	[111]
HepG2	Pretreated with chrysin for 2 h	Sensitized the apoptosis induced by cisplatin and camptothecin	[112]
HepG2 cells	1 to 15 μM 48 h	Stopped the SubG0 phase	[113]
HepG2 cells	24 h	Enhanced TRAIL-mediated apoptosis in hepatocellular carcinoma cell line HepG2	[114]
BEL-7402 cells	Ptreated with 10 lM of chrysin prior to 48 h incubation	Reduced anticancer drug resistance by down-regulating Nrf2 signaling pathway	[115]
Xenograft mice	15, 30, 60 mg/kg for 10 days	Increased caspase-3 activity, inhibited VEGF creation, and inhibited angiogenesis	[116]
HepG2 cells	10, 20, and 40 μmol/L for 2 h	Accelerated the apoptosis induced by TNF-α, the down-regulation of apoptosis inhibitory protein cFLIP-L, which is regulated by NF-κB and augmented by TNF-α	[117]
HepG2 SMMC-7721	2.5 to 40 μM 48 h	Inhibited hepatoma cells growth and induces apoptosis in a dose-dependent manner.	[118]
DEN-induced early hepatocarcinogenesis in rats	250 mg/kg three times weekly for 3 weeks	Activated p53-mediated apoptosis during early hepatocarcinogenesis	[119]
HepG2 Human hepatocytes	1-50 µM 72 h	Induced UGT1A1 expression was minimal in human hepatocytes treated with chrysin compared with that in HepG2 cells	[120]
HepG2	10 and100 µg/ml	Activated UDGPT and/or inhibition of sulfotransferase in 2-amino-1-methyl-6-phenylimidazo[4,5-]pyridine (PhIP)-HepG2	[121]
HepG2 cells	Chrysin-NS 0, 0.1, 1, 10 and 100 μg/mL	Inhibited the proliferation	[122]

## LIST OF ABBREVIATIONS

MMP: Matrix Metalloproteinases
HCC: Hepatocellular Carcinoma
HFF: Human Foreskin Fibroblast
LPS: Lipopolysaccharide
